# Association Between Phase Coupling of Respiratory Sinus Arrhythmia and Slow Wave Brain Activity During Sleep

**DOI:** 10.3389/fphys.2018.01338

**Published:** 2018-09-25

**Authors:** Kyuichi Niizeki, Tadashi Saitoh

**Affiliations:** Department of Biosystems Engineering, Graduate School of Science and Engineering, Yamagata University, Yamagata, Japan

**Keywords:** autonomic nervous system, slow wave sleep, respiratory sinus arrhythmia, phase coherence, sleep cycle

## Abstract

Phase coupling of respiratory sinus arrhythmia (RSA) has been proposed to be an alternative measure for evaluating autonomic nervous system (ANS) activity. The aim of this study was to analyze how phase coupling of RSA is altered during sleep, in order to explore whether this measure is a predictor of slow wave sleep (SWS). Overnight electroencephalograms (EEG), electrocardiograms (ECG), and breathing using inductance plethysmography were recorded from 30 healthy volunteers (six females, age range 21–64, 31.6 ± 14.7 years). Slow wave activity was evaluated by the envelope of the amplitude of the EEG δ-wave (0.5–4 Hz). The RSA was extracted from the change in the R-R interval (RRI) by band-pass filter, where pass band frequencies were determined from the profile of the power spectral density for respiration. The analytic signals of RSA and respiration were obtained by Hilbert transform, after which the amplitude of RSA (A_RSA_) and the degree of phase coupling (λ) were quantified. Additionally, the normalized high-frequency component (HF_n_) of the frequency-domain heart rate variability (HRV) was calculated. Using auto- and cross-correlation analyses, we found that overnight profiles of λ and δ-wave were correlated, with significant cross-correlation coefficients (0.461 ± 0.107). The δ-wave and HF_n_ were also correlated (0.426 ± 0.115). These correlations were higher than that for the relationship between δ-wave and A_RSA_ (0.212 ± 0.161). The variation of λ precedes the onset of the δ-wave by ~3 min, suggesting a vagal enhancement prior to the onset of SWS. Auto correlation analysis revealed that the periodicity of λ was quite similar to that of the δ-wave (88.3 ± 15.7 min vs. 88.6 ± 16.3 min, λ-cycle = 0.938 × δ-cycle + 5.77 min, *r* = 0.902). These results suggest that phase coupling analysis of RSA appears to be a marker for predicting SWS intervals, thereby complementing other noninvasive tools and diagnostic efforts.

## Introduction

During sleep, two different states of sleep, non-rapid eye movement (NREM) and rapid eye movement (REM) sleep, alternate cyclically with a period of 80–120 min (Merica and Gaillard, 1985). Slow wave sleep (SWS) is a deep NREM sleep characterized by the presence of δ-waves in electroencephalogram (EEG) recordings, which also oscillate with a similar period (Aeschbach and Borbély, 1993). The SWS stage is considered to be the most restorative interval of sleep for both the brain and body (National Sleep Foundation., 2006). During this period, human growth hormone is released as the body rejuvenates (Born et al., 1988; Brandenberger et al., 2005). A lack of SWS may cause increased daytime sleepiness and lead to medical problems, such as obesity (Taheri et al., 2004), hypertension (Fung et al., 2011), diabetes (Tasali et al., 2008), and memory disruption (Gais and Born, 2004). Thus, increased time spent in SWS is associated with better sleep quality, while a reduced amount of SWS is generally regarded as an indicator of poor sleep. In this context, accurately detecting the amount of SWS occurring during sleep is of importance and interest.

Sleep stages and patterns are identified based on polysomnography (PSG), which requires multiple physiological recordings of eye movements, muscle activity, and heart rhythm, including EEG activity. A well-trained expert often conducts manual sleep stage scoring according to clinical standards proposed by the American Academy of Sleep Medicine (Iber et al., 2007). Because this procedure is generally a tedious and time-consuming task, a more convenient approach that can facilitate self-monitoring at home is becoming increasingly necessary for a variety of reasons, such as a growing population of older adults for whom health conditions must be regularly monitored. One approach for the assessment of sleep quality other than PSG is the analysis of fluctuation in the autonomic nervous system (ANS) during sleep using cardiorespiratory signals (Cabiddu et al., 2012; Penzel et al., 2016). On the basis of heart rate variability (HRV) analysis, sleep-stage dependent modulation of ANS activity has been demonstrated. Studies conducted using spectral analysis of HRV to evaluate ANS control during wakefulness and different sleep stages have shown that the transition from wake to NREM sleep is associated with a gradual increase in parasympathetic modulation, which is expressed as an increase in the high-frequency (HF) power of HRV and a decrease in the low- frequency (LF) to HF power (LF/HF) ratio thought to reflect sympathovagal balance (Shinar et al., 2006; Tobaldini et al., 2013). On the contrary, a shift in sympathovagal balance toward vagal withdrawal and sympathetic predominance (i.e. an increase in LF/HF) has been reported to occur in the transition from NREM to REM sleep (Berlad et al., 1993; Otzenberger et al., 1998; Miyashita et al., 2003; Cabiddu et al., 2012). Such changes in indirect measurements of ANS activities do not contradict with changes in blood pressure and heart rate observed during sleep (Vaughn et al., 1995; Burgess et al., 2001). Moreover, previous studies have reported an inverse coupling between SWS activity and the normalized LF power of HRV (Brandenberger et al., 2001) and a significant decrease in the LF/HF ratio during SWS period (Shinar et al., 2006). However, there is a continuing debate over the validity of the use of the LF power of HRV as an index of sympathetic nerve activity (Randall et al., 1991; Goldstein et al., 2011). Also, the claim that the LF/HF ratio is thought to reflect sympathovagal balance has recently been called into question (Billman, 2013).

In addition to HRV analysis, cardiorespiratory phase synchronization has recently become of great interest for investigating the association between ANS activity and sleep. It has been shown in healthy subjects that phase synchronization between respiration and heartbeat occurs for the largest percentage of time during deep sleep, while the percentage of time with synchronization is smallest during REM sleep (Bartsch et al., 2012). This synchronization has been shown to occur independently of the amplitude of heart rate modulation by respiration, i.e., respiratory sinus arrhythmia (RSA). However, we previously observed in humans that the degree of phase coupling between RSA and respiration was also modulated by changes in ANS activity (Niizeki and Saitoh, 2012), where mental stress induced an incoherent phase lag with respect to breathing, resulting in a decrease in the phase coherence (λ) between RSA and respiration, in addition to a decrease in the amplitude of RSA. λ has been shown to be positively correlated with the HF component of the frequency-domain measurements of HRV and negatively correlated with sympathovagal balance (Niizeki and Saitoh, 2012). Since RSA is mediated predominantly by the modulation of cardiac vagal efferent activity (Eckberg, 2003), this observation suggests that the dynamic transfer of vagal-cardiac nerve traffic is altered by phasic vagal modulation elicited by mental stress, which leads to breath-to-breath variations in the latency of RSA. λ has also been suggested to be a sensitive measure for evaluating cardiac autonomic profiles during feeding behavior compared to the frequency-domain HRV indices (Niizeki and Saitoh, 2016). Based on these findings, it was expected that the phase coupling of RSA could act as an alternative indirect measure for evaluating ANS activity. We assumed that if SWS induced strong cardiorespiratory coupling (i.e., an increase in λ) as a reflection of cardiac parasympathetic activation and sympathetic inhibition during SWS period, λ could be used as an indicator for detecting SWS intervals during sleep. The aim of the present study was to examine how the coherent oscillation of RSA is altered during nocturnal sleep and to evaluate the temporal relationship between brain slow wave activity and λ.

## Methods

### Subjects

Thirty subjects (six females), age range 21–64 (31.6 ± 14.7) years with normal body mass indices (22.3 ± 2.5 kg/m^2^) were recruited for participation in the study. Among them 25 were below the age of 32 years old (four females) and 5 were above 60 years old (two females). All were normotensive non-smokers and none had a history or current symptoms of psychopathology or neurology known to influence sleep or the ANS. The experimental protocol was approved by the Yamagata University Institutional Ethics Committee and the study conformed to the Declaration of Helsinki (approval number: 28–17). All subjects were given written and verbal instructions as to the nature of the recordings and experimental procedures, and were required to sign a consent form.

### Instrumentation

Experiments were carried out in a quiet room with the temperature maintained at 22–24°C. On the night of data measurement, subjects arrived at the laboratory at ~10:00 p.m., where devices were prepared to record electrocardiogram (ECG), electroencephalogram (EEG), and respiratory inductance plethysmography (RIP). Subjects abstained from drink containing caffeine or alcohol, and did not perform strenuous exercise during the12 h prior to arriving at the laboratory. Ag-AgCl disposable electrodes were attached to the subject's chest and head for obtaining unipolar ECG and frontopolar EEG (Fp1/A1), respectively. The skin was gently abraded with polish gel (SkinPure; Nihon Kohden) and cleaned with alcohol to reduce skin electrode impedance. Subjects wore a thoracic belt for RIP (z-RIP; Pro-Tech Services, Mukilteo, WA, USA) in order to measure breathing movements. The belt was positioned around the rib cage at the level of the axilla. Three Actiwave devices (CamNtech Ltd, Cambridge, UK) were used for continuous ECG, EEG, and RIP recordings. They consisted of miniature solid-state biosignal recorders worn discreetly on the body without the need for a recorder or lengthy wires. The sampling frequency of the ECG, EEG, and RIP were set at 512, 128, and, 32 Hz, respectively, with 10-bit resolution. The start time for the recording was set to occur between 11:00 p.m. and 12:00 a.m., according to the subject's usual bed time. Subjects were instructed to remove the electrical wire connecting the disposable electrodes to the ActiWave device immediately after wakening on the following morning in order to accurately record the time spent in bed. Signals recorded by the ActiWave devices were transferred via European Data Format to LabChart 8 analysis software (ADInstruments, Sydney, NSW, Australia), and then exported to text files for analysis.

### Sleep EEG data processing

The detection of slow waves (δ-wave) was performed using a bandpass filter available in the LabChart software. The filter implemented in this software is a zero-phase-lag Finite Impulse Response type filter with a Kaiser window (β = 6). The passband frequencies were set at 0.5–4 Hz for the extraction of δ-wave data (Tasali et al., 2008). The temporal amplitude envelope of the extracted δ-wave was then computed based on analytic signal. An analytic signal [ζ(*t*)] was constructed from a real signal of δ-wave [*s*(*t*)] and its Hilbert transform [*s*_*H*_(*t*)] as $\zeta \left(t\right)=s\left(t\right)+is_{H}\left(t\right)=\upsilon \left(t\right)e^{i\varphi \left(t\right)}$, where υ(*t*) and ϕ(*t*) are instantaneous amplitude and instantaneous phase, respectively. The amplitude-enveloped time series of υ(*t*) were then effectively low-pass filtered (2nd order Butterworth, < 1.0 Hz) into 20 s windows and averaged by applying a median filter (2-min moving windows). This temporal evolution of the amplitude envelope of the δ-wave served as the signal for correlation analysis. In addition, in order to visualize the frequency contents of EEG signal, spectral decomposition was accomplished by short-time Fourier transform using a section of 2048 samples (a 32-s time window, shifting every 2 samples) with a Hanning window applied.

### ECG and RIP data processing

The beat-to-beat R-R intervals (RRIs) were derived as the duration between successive ECG R peaks in a cardiac cycle. A median filter was applied to remove the wandering baseline artifact which can result from subject movement. To obtain RRIs with equidistant time steps, the beat-to-beat RRI was resampled at a frequency of 10 Hz using the Spline interpolation method. Before resampling, the calculated RRIs were visually inspected for artifacts or inaccurate R wave detection and corrected by manual editing with interpolation. To obtain a time series for the breathing movement at corresponding sampling times, stored RIP signals were also resampled at 10 Hz. The RIP signal was then filtered by low-pass Butterworth filter (< 1 Hz) to exclude gross body movements that can obscure the respiratory movements.

### Extraction of RSA

The time series of the RRI was further band-pass filtered to determine the RSA oscillation. Before construction of the band-pass filter, the frequency characteristics of the breathing were obtained using a continuous Wavelet transform (CWT). The results were then used to produce a passband frequency for extraction of the RSA. The CWT of a data set *s*(*t*) for a chosen wavelet function ψ(*t*) is defined as the convolution of *s*(*t*) with a scaled and translated version of ψ(*t*):

$$\begin{array}{l} W\left(a,t_{0}\right)=\frac{1}{\sqrt{a}}\int_{-\infty }^{\infty }s\left(t\right)\psi^{*}\left(\frac{t-t_{0}}{a}\right)dt \end{array}$$

**Figure 1:**
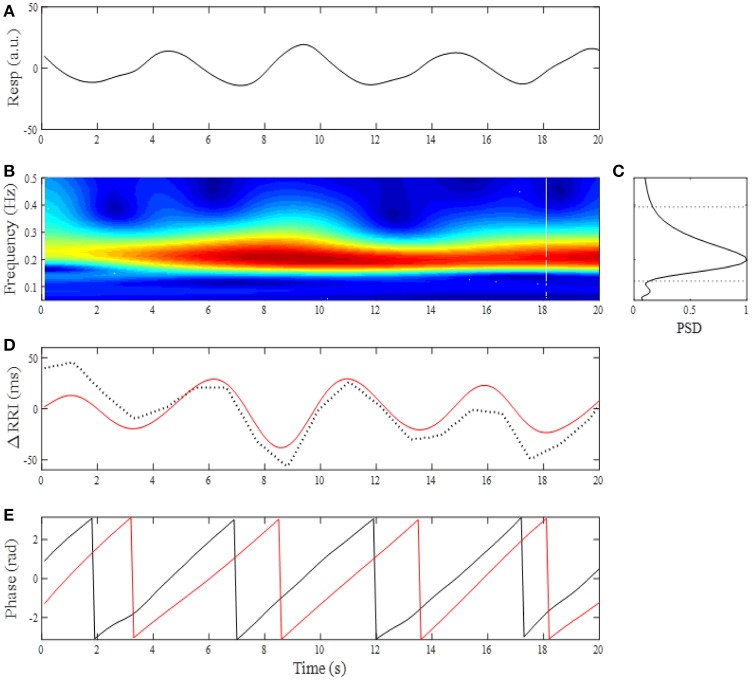
**(A)** Changes in breathing trace (Resp, upward deflections denote expiration) for a 20-s time window. **(B)** Time-frequency representation by CWT for breathing. **(C)** Mean power spectral density (PSD) profile in the normalized form. The corner frequencies of passband filter for determining RSA were estimated by the frequencies where PSD reduced to 20% of its maximum power (horizontal dotted lines). **(D)** Change in RRI (dotted curve) and extracted RSA (red curve). **(E)** Instantaneous phases for breathing (black line) and RSA (red line).

Where, *a* is a time scale parameter, *t* is time, and (^*^) refers to the complex conjugate (Daubechies, 1990). We used a complex Morlet wavelet basis function to obtain a CWT of the respiratory signal. Before calculation of the CWT of the respiratory signal, the average value of the respiratory signal was subtracted, normalizing its mean value to zero. The power spectral density (PSD) of the CWT was obtained as the absolute value of *W*(*a*,*t*). An example of the analysis is shown in Figure 1, where a breathing trace for a 20-s window (Figure 1A) and its time-frequency representation (TFR, Figure 1B) are shown. At each moment of time sampled, the maximum of the local spectrum provides an instantaneous respiratory frequency (f_R_), which is determined by finding, at each timepoint, the most dominant peak in the TFR amplitude. By ensemble averaging the TFR amplitudes at each time window, a mean PSD profile was obtained (Figure 1C) and then low- (f_RL_) and high (f_RH_) corner frequencies of band-pass filter were determined by the frequencies where the PSD reduced to 20% of its maximum power (Figure 1C, dotted horizontal lines). With the use of the passband frequencies, we adapted a 3rd order zero-phase Butterworth band-pass filter to the RRI time series for extraction of the RSA.

### Phase coupling of RSA

RSA is a phenomenon characterized by RRI fluctuations in accordance with respiration, by which the RRI is shortened during inspiration and prolonged during expiration. There is a phase lag between RSA and respiration, with the instantaneous phase of respiration (ϕ_resp_) slightly preceding the instantaneous phase of RSA (ϕ_RSA_). Although the exact relationship between ϕ_resp_ and ϕ_RSA_ depends on f_R_, phase difference between ϕ_resp_ and ϕ_RSA_ remains constant throughout respiratory cycle as long as f_R_ remains stable (see Figure 1E). This is because a coupling between respiration and neurons involved in the generation of RSA tends to keep the phase difference at a constant value (phase coupling). Although the physiological origins and mechanism of RSA are still a matter of debate, RSA has been considered to be a consequence of direct central respiratory modulation of the cardiac vagal activity and reflex responses from pulmonary and cardiovascular receptors mediated through afferent vagal nerve fibers (Berntson et al., 1997; Eckberg, 2003). Thus if dynamic transfer of cardiac-vagal nerve traffic is modulated by intrinsic or extrinsic factors, this modulation affects phase stability of RSA oscillations, which in turn leads to variations in the degree of phase coupling of RSA. The phase coupling of RSA was evaluated by the phase coherence (λ) between respiration and RSA. From the oscillatory signals of respiration and RSA, an analytic signal was constructed in each signal from a real signal and its Hilbert transform (Figure 1E). The phase evolutions at each time were then used to compute λ between ϕ_resp_ and ϕ_RSA_. Averaging the instantaneous amplitude (envelope) of RSA in a time-window gives the mean amplitude of RSA (A_RSA_). The time-dependent λ and A_RSA_ are defined as: $\lambda \left(t_{k}\right)={\left|\frac{1}{N}\overset{k+N/2}{\underset{j=k-N/2}{\sum }}e^{i\psi \left(t_{j}\right)}\right|}^{2}$, $A_{RSA}\left(t_{k}\right)=\frac{1}{N}\overset{k+N/2}{\underset{j=k-N/2}{\sum }}|\upsilon \left(t_{j}\right)|$, where ψ(*t*) = [ϕ_resp_(*t*) - ϕ_RSA_(*t*)] mod 2π, υ(t) is the instantaneous amplitude of RSA, and N denotes the number of consecutive data samples to be considered in the computation. The λ and A_RSA_ values were calculated from 200-point windows (20 s) with a 100-point (10 s) sliding window, then their trends were obtained by applying a 12-point (2-min) median filter.

### Frequency domain analysis of HRV

In order to analyze the HRV in the frequency-domain, the spectral component was identified on the HRV spectrum in the high-frequency (HF, frequency range: f_RL_-f_RH_ Hz) band using a CWT. Then the HF component of the HRV spectrum was computed by the integration of the spectral component, in order to estimate the level of the vagal branch of the ANS to the heart. The power spectrum for the HF component was expressed in normalized units as HF_n_ = HF/TF, where TF denotes the total frequency component (0.04–f_RH_ Hz) of the HRV spectrum. The HF_n_ index was Spline interpolated into 10-s interval data and then its trend was obtained by applying a 2-min median filter.

### Auto correlation and cross-correlation analyses

It is well known that NREM and REM sleep continue to alternate through the night in a cyclic fashion characterized by a sequence starting with light sleep, followed by SWS and REM sleep. This sleep structure repeats for a total of 4–6 cycles in healthy sleep (Merica and Gaillard, 1985). Given that the change in δ-wave activity oscillates with a similar period (Aeschbach and Borbély, 1993), we were interested to know whether the λ, A_RSA_, and HF_n_ indices would show the same oscillation with the δ-wave. We estimated the sleep cycle from the alternating change in δ-activity using an auto- correlation function (ACF). The ACF gives a maximum value of 1 at zero lag and also a peak for lag corresponding to the time of an integer multiple of the fundamental oscillation. The cyclic period of the δ-wave was determined as the duration (*D*_i_) between the successive times showing peaks in the ACF. Because the ACF is symmetrical about lag zero, positive lag was taken into consideration when estimating the cyclic period. If multiple peaks were present, the mean duration ($\bar{D}$) was calculated as follows:$\bar{D}=1/n_{p}\overset{n_{p}}{\underset{i=1}{\sum }}D_{i}/i$, where *n*_*p*_ is the number of peaks detected. The ACF was also calculated in the λ, A_RSA_ and HF_n_ to estimate their overnight periodicity. If the ACF peak was indistinct, the dominant frequency was determined from the periodogram of the ACF. The PSD of the ACF was obtained from a FFT using zero padding and a Hanning window with a frequency resolution either 1.22 × 10^−5^ Hz (window length of 8192) or 2.44 × 10^−5^ Hz (window length of 4096). Then, the PSD of the ACF was normalized by the maximum power spectrum. In order to quantify the periodicity of the ACF, we defined the oscillation strength (OS) as the ratio between the magnitude of dominant oscillation frequency and the total magnitude of the ACF spectrum as follows:$OS=\frac{\overset{1.25f_{p}}{\underset{0.75f_{p}}{\int }}p\left(f\right)df}{\overset{f_{U}}{\underset{f_{L}}{\int }}p\left(f\right)df}$, where *p*(*f*) is the estimated PSD of frequency *f* in the FFT-computed spectrum, *f*_*p*_ is the peak frequency of ACF corresponding to the sleep cycle, and *f*_*U*_ and *f*_*L*_ are the upper and lower frequencies of oscillations of the ACF, respectively. We set *f*_*U*_ and *f*_*L*_ to 0.0008 Hz (~21 min cycle) and 0.00008 Hz (~208 min cycle), respectively, as these frequency bands cover the span of frequencies pertinent to the ACF spectral peak observed in the present study. Because of the spectral spread, the peak frequency power was calculated by integration in the frequency range of 75–125% of *f*_*p*_. The OS was regarded as the strength of the dominant oscillation frequency corresponding to the sleep cycle relative to all frequencies in the spectrum. As the OS increases, the ACF oscillation with the period of the sleep cycle becomes clearer.

To quantify the degree of association between the brain δ-wave and the autonomic indices of λ, HF_n_, and A_RSA_, a cross-correlation function (CCF) was calculated for each subject for the entire night of sleep (Entire). The CCF was normalized with respect to the square root of the product of the auto-correlation of both bivariate signals at a zero time lag. The maximal CCF (CCF_max_) was used to evaluate the strength of concordance between the two rhythms. The latency (time delay, T_d_) of the correlation was defined as the difference between the time showing the CCF_max_ and zero lag time. The auto- and cross-correlation analyses were also separately applied to the first (F3_4) and last three-quarters (L3_4) of the sleep period to investigate whether the profiles of auto- and cross correlation depend on the elapsed time of sleep, since it has been shown that SWS is most prominent in the first half of the night and diminishes during the latter half of the night. All data analyses were performed in Matlab (MathWorks, Natick, MA, USA) using custom scripts.

### Statistics

Results are presented as mean±standard deviation. Regions where the amplitude of δ-activity was above the mean amplitude of δ-activity in the entire night of sleep were identified as high δ-wave activity segments. Then, each segment containing consecutive high- and low δ-wave activity was identified as one sleep cycle, by visual inspection (see Figure S1). The time courses for the λ, HF_n_, and A_RSA_ were aligned with the respective T_d_ values observed in the CCFs with δ-wave. The mean values for the amplitude of the δ-wave, λ, HF_n_, A_RSA_, f_R_, and range of f_R_ (f_RH_-f_RL_) over the segments showing high and low δ-wave activity were calculated separately during 1–3 sleep cycles for each subject and then averaged to obtain group mean values. Inter-indices comparisons in the OS and the differences in the CCF_max_ and T_d_ in the inter-relationships of indices of δ-wave, λ, HF_n_, and A_RSA_ were evaluated by one-way analysis of variance (ANOVA) during the entire night, as well as the F3_4, and L3_4 sleep periods. When a significant *F* value was detected, it was further examined using a Bonferroni *post hoc* test to assess pairwise comparisons for the respective variables and the various analysis periods of the night (i.e., Entire, F3_4, and L3_4). Pearson's product-moment correlation coefficient was used to estimate the correlations of the correlation metric. All statistical tests were 2-sided, and a *P* value < 0.05 was judged to be statistically significant. Statistical analyses were performed using the software FreeJSTAT (Version 22.E).

## Results

**Figure 2:**
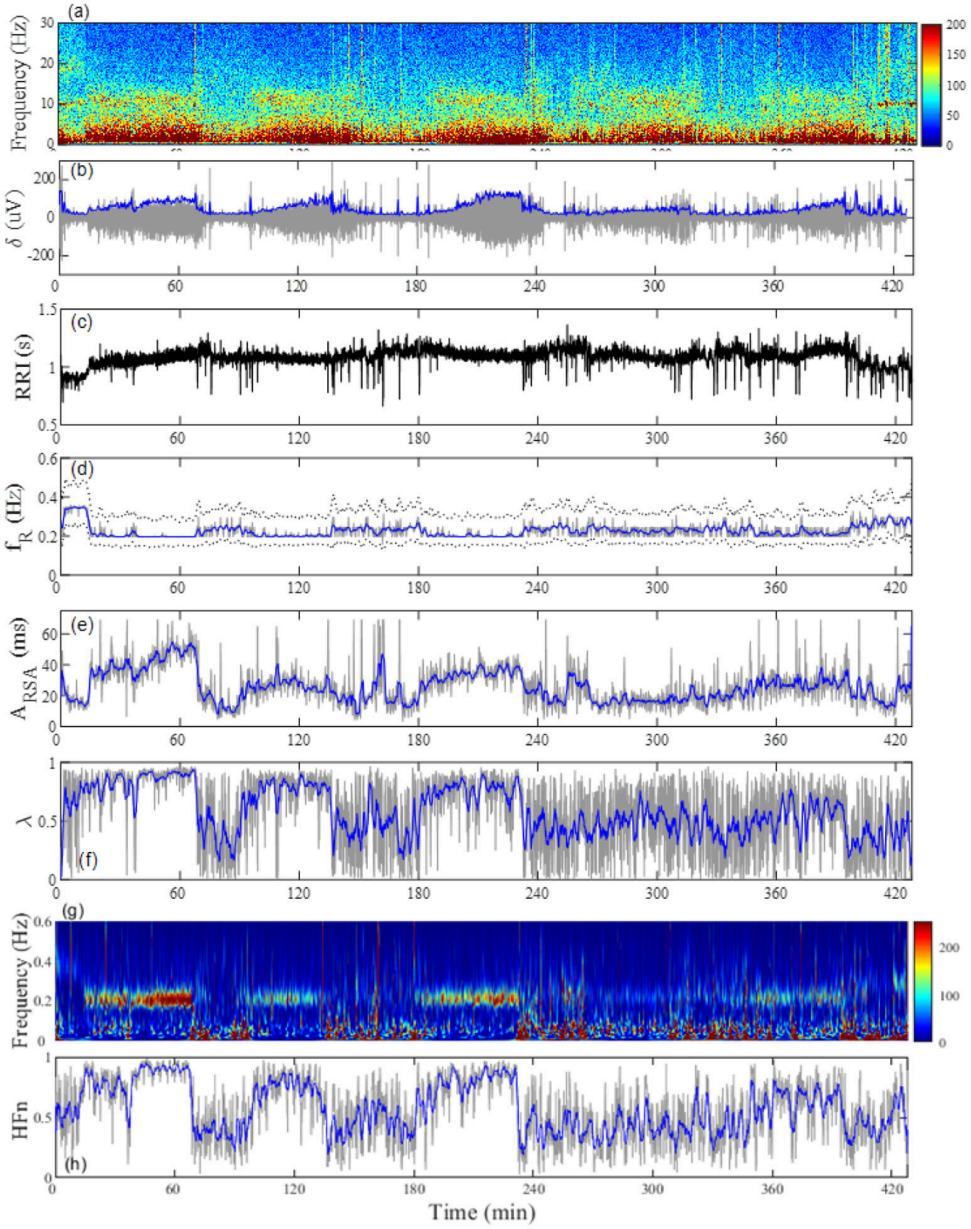
Representative profiles of the **(A)** EEG frequency decomposition, **(B)** extracted EEG δ-wave, **(C)** RRI, **(D)** breathing frequency (f_R_), **(E)** amplitude of respiratory sinus arrhythmia (A_RSA_), **(F)** phase coherence (λ) between RSA and respiration, **(G)** time-frequency representation (TFR) for the change in RRI by using a continuous wavelet transform, and **(H)** normalized high-frequency component (HF_n_) of RRI fluctuation estimated from TFR in a 24-year-old man. The gray line in **(B)** indicates the amplitude of the EEG δ-wave and the blue line depicts the envelope. The dotted lines in **(D)** indicate the full width at 20% maximum of the power spectral density for breathing, which was used as the passband frequency for determination of RSA. The gray lines in **(D–F,H)** are respective values estimated from 20-s windows and the blue lines indicate general trends obtained by applying a median filter (2-min moving window).

**Figure 3:**
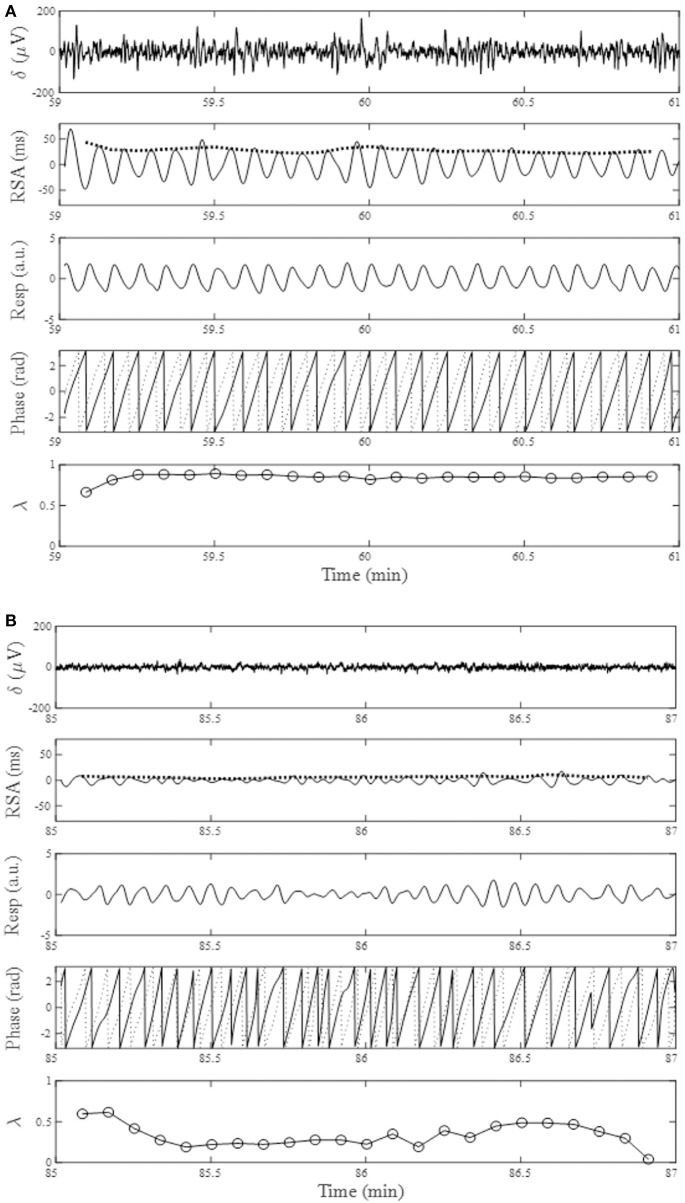
From the top, changes in EEG δ-activity (δ), RSA, breathing trace (Resp in arbitrary unit; upward deflections denote expiration), instantaneous phases for RSA (solid trace) and breathing (dotted trace), and phase coherence (λ) calculated from 20-s windows with a sliding window of 10-s for a 2-min portion around increased EEG δ-activity (**A**, 59–61 min) and decreased EEG δ-activity (**B**, 85–87 min). Bold dotted curve in the RSA panel indicates A_RSA_. Data presented are form the same subject shown in Figure 2.

The average time in bed for the 30 subjects was 421±32 min. Example of the overnight profiles of the brain δ-wave, R-R interval (RRI), respiratory frequency (f_R_), amplitude of RSA (A_RSA_), and λ, as well as the normalized HF index (HF_n_) for an individual subject are shown in Figure 2. The δ-wave showed clear cyclic changes, particularly in the first half of the night (Figure 2B). At the portions where δ-activity decreased such as the periods between 80–90 min and 160–180 min, f_R_ appeared to increase (Figure 2D). The trend of the changes in A_RSA_ and λ appeared similar to the change in δ-wave activity (Figures 2E,F). The time-frequency analysis of HRV using the CWT revealed that the change in the pattern of HF_n_ (Figure 2H) was almost paralleled by that in A_RSA_ and λ. When we looked at the portion where δ-wave activity increased (59–61 min in Figure 2), the amplitude of the RSA oscillation was large and the phase relationship between RSA and respiration was quite stable, and therefore λ comes close to unity (Figure 3A). On the other hand, when δ-activity decreased (85–87 min in Figure 2), the amplitude of RSA oscillation was small and the phase relationship between RSA and respiration is relatively unstable, yielding a low λ value (Figure 3B). Although we did not measure electro-oculogram activity or chin muscle tone, this portion was assumed to be the REM stage judging from the visual inspection of the frequency contents of EEG spectrogram (Figure 1A), where low-amplitude, mixed-frequency EEG activity was present. In addition, increased and irregular breathing patterns were seen at this period (Figure 2D).

**Figure 4:**
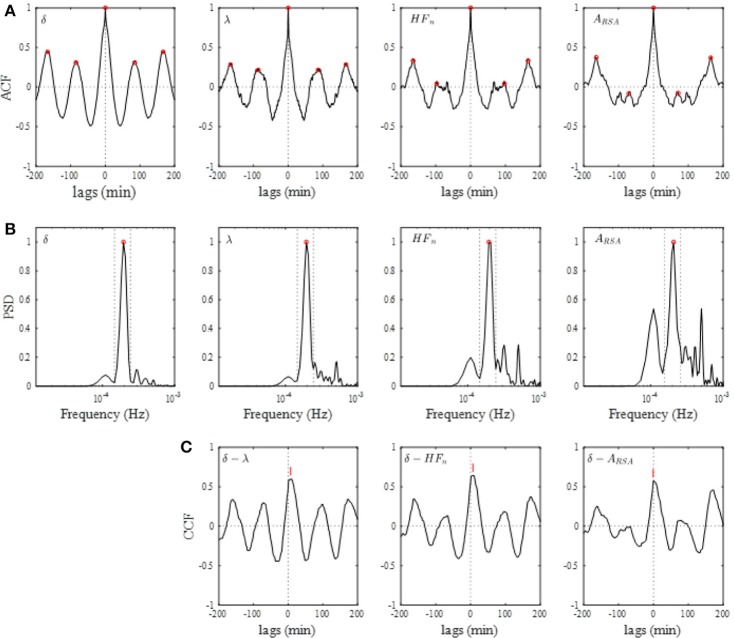
**(A)** Auto-correlation functions (ACFs) of brain δ-wave, λ, normalized high-frequency component of HRV (HF_n_), and amplitude of RSA (A_RSA_), calculated from the entire night of sleep. Open red circles indicate peaks of ACF. **(B)** Normalized power spectral densities (PSDs) for the respective ACF. Open red circle, detected maximal peak; vertical dotted lines, ± 25% of the peak frequency. **(C)** Cross-correlation functions (CCFs) between δ-wave and λ, between δ-wave and HF_n_, and between δ-wave and A_RSA_. The lag time at which CCF attains its maximum is indicated by a red vertical bar in each CCF.

**Figure 5:**
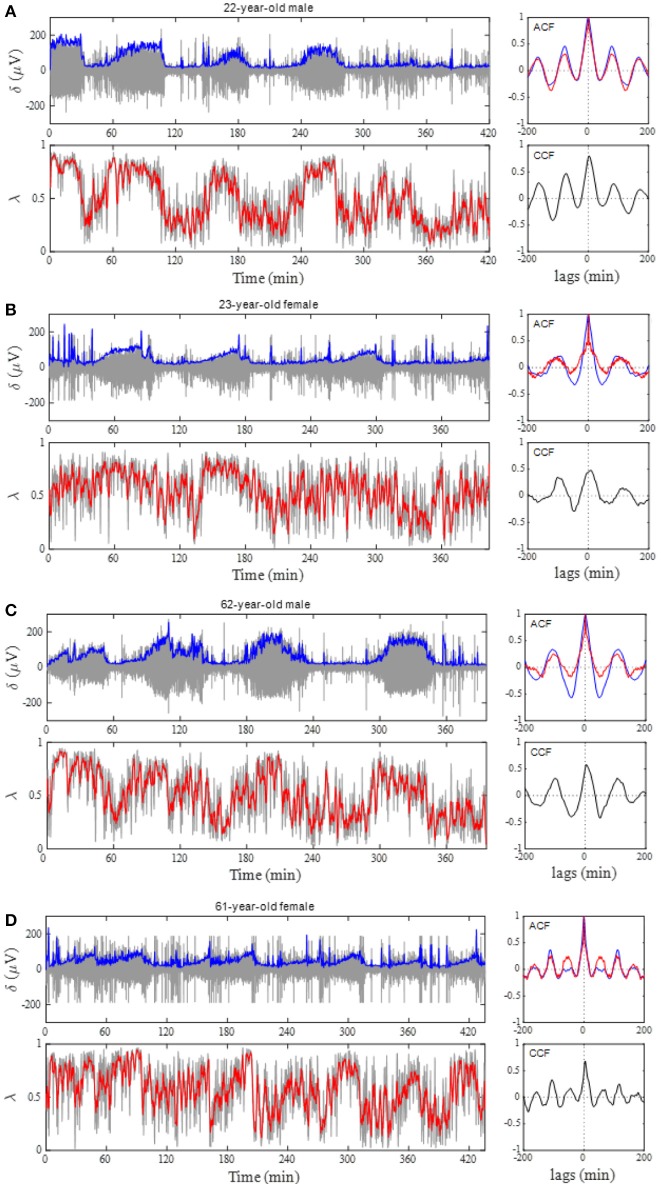
Phase coupling analyses for male and female subjects of different ages. **(A)** 22-year-old man, **(B)** 23-year-old woman, **(C)** 62-year-old man, and **(D)** 61 year-old woman. Profiles of the EEG δ-wave and phase coherence (λ) for the whole night are shown, together with their respective autocorrelation function (ACF) and cross-correlation function (CCF) between these two signals. Blue and red curves in the ACF panel indicate ACF for the δ-wave and λ, respectively. Depicted in the gray and blue in δ-wave panel show an extracted δ-wave and its envelope, respectively. Computed λ from 20-s windows shows in gray line and the red indicates general trend obtained by applying a median filter (2-min moving window).

Figure 4A shows the results of the auto-correlation analysis of δ-wave and λ as well as A_RSA_ and HF_n_ indices for the same subject presented in Figure 2. Clear oscillation was seen in the ACF for the δ-wave with maxima spaced at ~83 min, whereas the profile of the ACFs for the λ, HF_n_ and A_RSA_ had apparent “ripples” in their ACF oscillations. This feature was supported by the PSD profiles of the ACFs obtained from the FFT. As can be seen in Figure 4B, there was a single prominent peak centered at about 0.0002 Hz (equal to a period of 83 min) in all PSD profiles. However, other oscillatory peaks existed in the PSD profile for the HF_n_ and A_RSA_. Therefore, the OS of HF_n_ (0.405) and A_RSA_ (0.283) were low compared to those for δ-wave and λ (0.662 and 0.525, respectively). Figure 4C shows the CCFs between the δ-wave and λ, δ-wave and HF_n_, and δ-wave and A_RSA_. All CCF attained maximum values (CCF_max_; 0.599, 0.651, and 0.574 for the relation of δ – λ, δ – HF_n_, and δ – A_RSA_, respectively) at time lags close to zero, but the CCF_max_ appeared at a slightly positive time lag in all of the relationships (red vertical bars in Figure 4C). Other examples of the profiles of EEG δ-wave and λ during the whole night for male and female subjects of different ages are shown in Figure 5, together with respective ACFs and the CCF between these two signals. Although the periodic pattern of the oscillations in the ACFs for the δ-wave and λ varied from individual to individual, a similar periodicity between these two signals was found.

Table 1 summarizes the average values of the amplitude of the δ-wave, λ, HF_n_, A_RSA_, f_R_, and frequency range of f_R_ (f_RH_-f_RL_) calculated separately in the segments for the high and low δ-wave activity on all subjects, for each sleep cycle. The λ, HF_n_, and A_RSA_ were consistently greater during the segments of high δ-wave activity than those of low δ-wave activity, throughout the sleep cycles. Judging from the range of f_R_, change in the variation of f_R_ increased during low δ-wave activity segments for each sleep cycle.

**Table 1:** Average values of the amplitudes of δ-wave, λ, HF_n_, A_RSA_, f_R_, and range of f_R_ calculated separately in the intervals of high and low δ-wave activity during 1–3 sleep cycles.

	**Sleep cycle**					
**Parameters**	**1**		**2**		**3**	
	**H**	**L**	**H**	**L**	**H**	**L**
δ-wave, μV	94 ± 25^**^	35 ± 7	85 ± 27^**^	32 ± 6	81 ± 23^**^	31 ± 6
λ	0.64 ± 0.12^**^	0.45 ± 0.13	0.60 ± 0.12^**^	0.40 ± 0.12	0.57 ± 0.14^**^	0.39 ± 0.11
HF_n_	0.65 ± 0.16^**^	0.51 ± 0.16	0.61 ± 0.19^**^	0.49 ± 0.16	0.62 ± 0.21^**^	0.52 ± 0.17
A_RSA_, ms	79 ± 56^**^	68 ± 52	82 ± 63^**^	75 ± 59	80 ± 64^*^	74 ± 58
f_R_, Hz	0.24 ± 0.03	0.24 ± 0.02	0.24 ± 0.02	0.24 ± 0.02	0.24 ± 0.02	0.24 ± 0.02
f_R_ Range, Hz	0.19 ± 0.03^*^	0.20 ± 0.03	0.18 ± 0.03^*^	0.20 ± 0.03	0.18 ± 0.03^*^	0.20 ± 0.03

*Values are mean ± SD*.

*H and L denote high and low δ-wave activity segments, respectively, discriminated by the mean amplitude of δ-wave over the entire night, i.e., H is the region where amplitude of the δ-wave was greater than the mean amplitude of the δ-wave over the entire night*.

*HF_n_, normalized high frequency component of heart rate variability; A_RSA_, amplitude of respiratory sinus arrhythmia; f_R_, respiratory frequency; f_R_ Range, range between the lower and upper corner frequencies where PSD for respiration reduced to 20% of its maximum power*.

** P < 0.01 and

* *P < 0.05 vs. L in each sleep cycle*.

**Figure 6:**
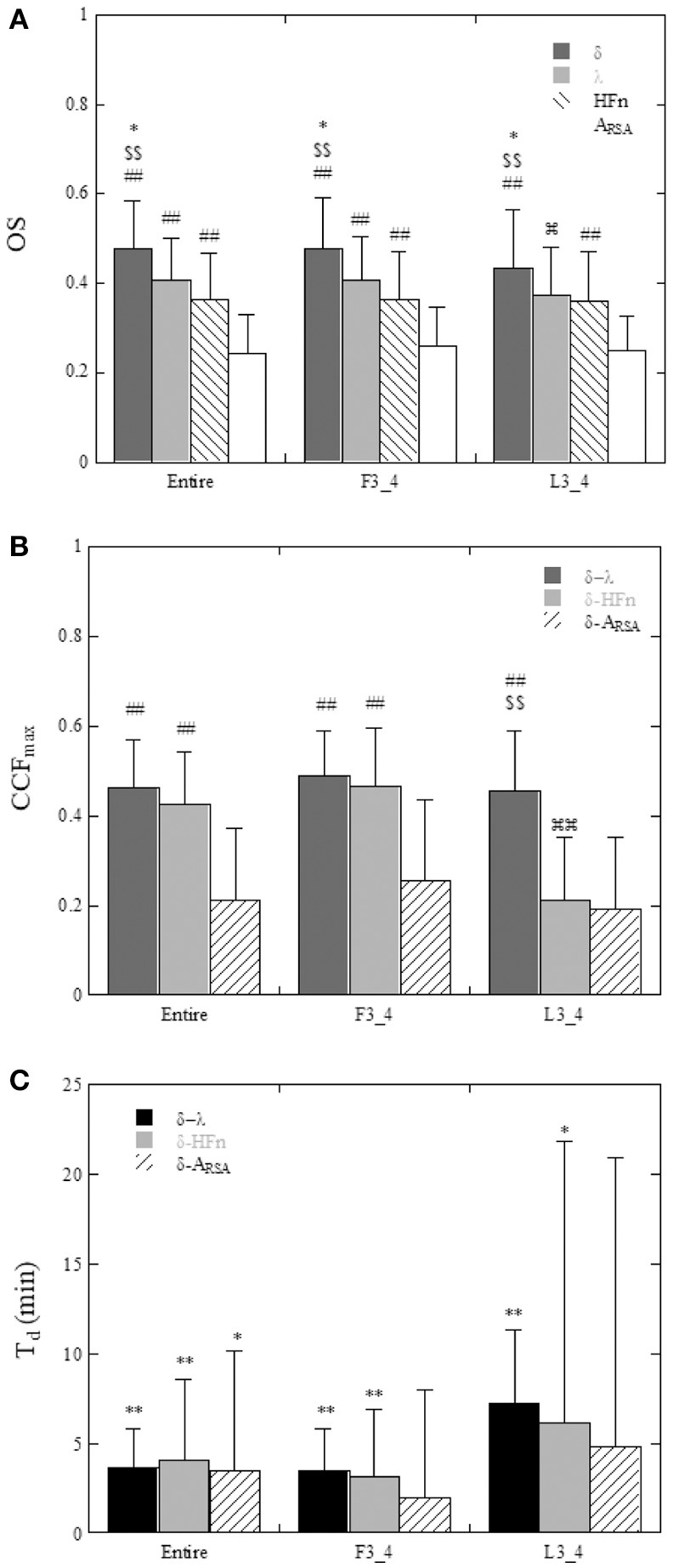
**(A)** Comparison of the oscillation strength (OS) of the ACF of δ-wave, λ, HF_n_, and A_RSA_ calculated for the entire night (Entire), the first three quarters (F3_4), and last three quarters (L3_4) of the sleep period. *P < 0.05 vs. λ, ^$$^*P* < 0.01 vs. HF_n_, ^##^*P* < 0.01 vs. A_RSA_, 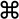
*P* < 0.05 vs. Entire and F3_4. **(B)** Maximum values of the cross-correlation function (CCF_max_) between δ-wave and λ, between δ-wave and HF_n_, and between δ-wave and A_RSA_. ^##^*P* < 0.01 vs. δ-A_RSA_, ^$$^*P* < 0.01 vs. δ-HF_n_, 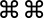
*P* < 0.01 vs. Entire and F3_4. **(C)** Time delay (T_d_) of the δ-wave from the changes in λ, HF_n_, and A_RSA_. Statistical comparison of T_d_ was performed with the null hypothesis of a zero time delay for each variable. *P < 0.05, **P < 0.01.

A comparison of the OS and CCF_max_ among the parameters in all subjects is shown in Figure 6. The OS of the δ-wave, λ, and HF_n_ were significantly greater (P < 0.01) than that of the A_RSA_ for the Entire and F3_4 sleep period (for Entire: 0.476 ± 0.108 for δ-wave, 0.407 ± 0.094 for λ, 0.364 ± 0.103 for HF_n_, and 0.242 ± 0.087 for A_RSA_, for F3_4: 0.477 ± 0.114 for δ-wave, 0.406 ± 0.097 for λ, 0.365 ± 0.106 for HF_n_, and 0.258 ± 0.089 for A_RSA_). The OS of the δ-wave and λ were reduced for the L3_4 period relative to the Entire or F3_4 sleep period (0.433 ± 0.130 for δ-wave and 0.372 ± 0.108 for λ in the L3_4). CCF_max_ for the correlations of the δ-wave – λ and δ-wave – HF_n_ were higher (*P* < 0.01) compared to the correlation of the δ-wave – A_RSA_ in the Entire and F3_4 sleep period (for Entire: 0.461 ± 0.107 for δ-wave – λ, 0.426 ± 0.115 for δ-wave – HF_n_, and 0.212 ± 0.161 for δ-wave – A_RSA_; for F3_4: 0.488 ± 0.100 for δ-wave – λ, 0.467 ± 0.128 for δ-wave – HF_n_, and 0.255 ± 0.180 for δ-wave – A_RSA_). Also, the CCF_max_ for the relationship between δ-wave and λ was consistently greater than that for the relationship between δ-wave and A_RSA_. The CCF_max_ between δ-wave and HF_n_ in the L3_4 sleep period was reduced compared to that calculated in the Entire and F3_4 sleep period.

The time delays (T_d_) are shown in Figure 6C. Using a *t*-test with the null hypothesis of zero time delay, all T_d_ were significantly different from zero except for the δ-wave – A_RSA_ relationship in the F3_4 and L3_4 sleep period. λ and HF_n_ showed a positive time delay in relation to the δ-wave, indicating that the change in EEG δ-activity lagged behind the changes in the λ and HF_n_. For the relationship between λ and the δ-wave in the Entire and F3_4 sleep period, the mean time delay was 3.6 ± 2.2 min and 3.5 ± 2.3 min, respectively.

**Figure 7:**
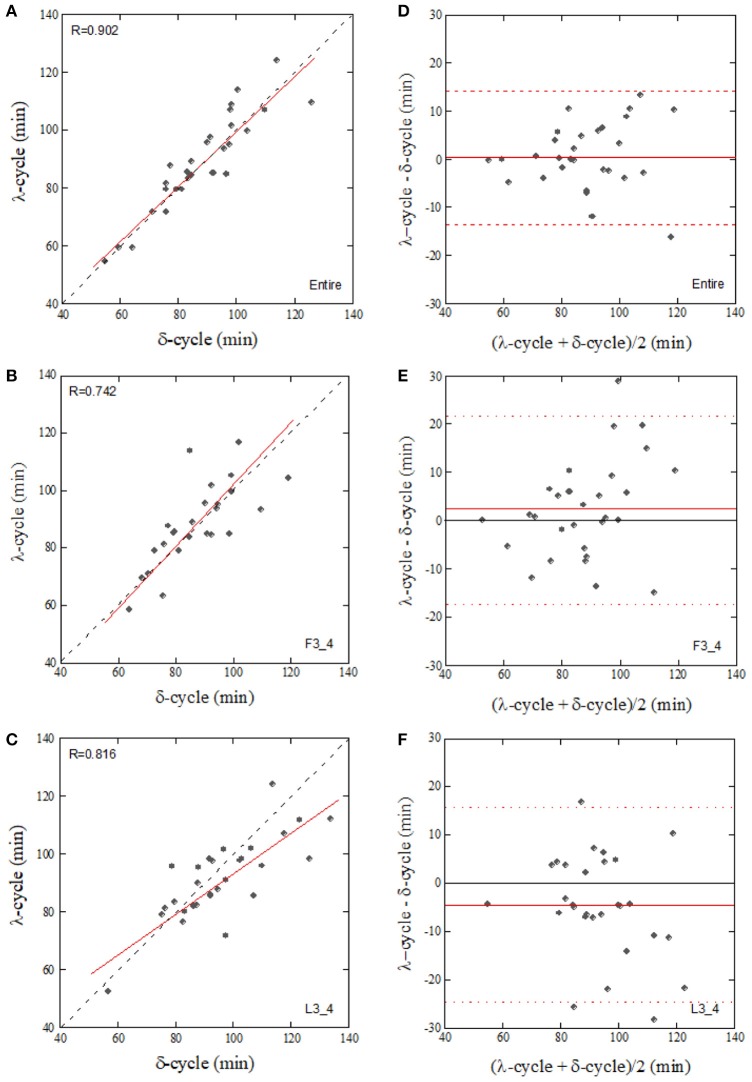
Correlation of the sleep-cycle estimated from δ-wave and λ ACFs calculated from the entire (**A**, Entire), first three-quarters (**B**, F3_4), and last three-quarters (**C**, L3_4) of the sleep period and their Bland-Altman plots **(D–F)**. The dotted and solid lines in **(A–C)** represent the identity line and linear regression line, respectively. Horizontal broken lines in **(D–F)**, upper and lower limits of agreement (+2 SD and −2 SD, respectively), Solid lines in **(D–F)**, mean value of the difference.

Since the CCF_max_ between δ-wave and λ showed a high value compared to that between the correlations of δ-wave – HF_n_ and δ-wave – A_RSA_, cyclic periods (sleep cycle) were determined from the ACFs for the δ-wave and λ, and then compared. Individual sleep cycles estimated from the λ ACF (λ-cycle) was plotted against that estimated from the δ-wave ACF (δ-cycle) for the Entire, F3_4, and L3_4 sleep periods (Figure 7). Regression line slopes produced from the relationship between δ-cycle and λ-cycle fell on similar trajectories in the Entire and F3_4 sleep periods (λ-cycle = 5.77 + 0.938 × δ-cycle for the Entire and λ-cycle = 1.77 + 1.01 × δ-cycle for the F3_4), however the slope of the regression line was lower for the L3_4 sleep period (λ-cycle = 23.9+ 0.699 × δ-cycle).

## Discussion

The present study demonstrated that cardiorespiratory coupling, assessed by the phase coherence (λ) between RSA and respiration, is associated with changes in δ-waves during nocturnal sleep, with variations in λ preceding variations in EEG δ-activity by ~3 min. Autocorrelation analysis revealed that the period of cyclical variation of the phase coupling of RSA is almost the same as the sleep cycle estimated from the oscillation of the δ-wave activity, as long as ACF is calculated from the entire or first three-quarters of the sleep period.

Our finding that elevated phase coupling of RSA precedes the onset of the increase in brain δ-activity is consistent with the previous observation by Thomas et al. (2014), who reported a tight temporal relationship between δ-wave power and cardiopulmonary coupling assessed by the magnitude coherence function between the HF power of HRV and respiration, with the changes in δ-wave power lagging behind the fluctuation in coherence by ~4 min. Since the HF power of HRV has been shown to be related almost exclusively to the oscillation of RSA, our results may add information supporting the notion that not only the magnitude of RSA but also the phase coupling of RSA is related to SWS activity. The existence of the onset latency of δ-wave activation to changes in HRV indices has been previously described in several studies (Otzenberger et al., 1997; Brandenberger et al., 2001; Jurysta et al., 2003; Long et al., 2015). However, the underlying mechanism involved in the latency of cortical activity is not fully understood. It is natural to postulate that neural pathways between cardiovascular medullary center and thalamocortical regions may mediate this interaction. Using simultaneous intra-cortical and intra-thalamic recordings, Magnin et al. (2010) demonstrated that cortical deactivation occurring at sleep onset lags behind thalamus deactivation by several minutes. This led to the suggestion that functional deafferentation of the cerebral cortex is a prerequisite to falling asleep, and is preceded by deactivation of the thalamus. Presumably, the brainstem regulating parasympathetic nervous function transmits signals to thalamic neurons via ascending tracts. Then, the cortex may be gradually entrained by the thalamus such that the EEG activity follows the cardiorespiratory phenomena. Interestingly, such a delay between vagal and cortical δ-wave activities has been shown to disappear in patients suffering from sleep apnea syndrome (Jurysta et al., 2006). Sleep apnea is known to induce sudden surges in sympathetic and vagal activity (Somers et al., 1995). This may induce suppression of the linkage between ANS activity and cortical activity. Under the assumption that elevated phase coupling of RSA (i.e., increase in cardiac vagal activity) leads to the initiation of the cortical down-state, this cortico-cardiorespiratory coupling may explain why sleep disorders, such as obstructive sleep apnea syndrome, are prone to develop sleep fragmentation. As has been documented, the majority of obstructive sleep apnea patients are accompanied by a sympathetic overactivity (Somers et al., 1995), which may disturb the cortico-cardiorespiratory coupling as well as coherent oscillation of RSA. Future experimental work using pharmacological autonomic blockade may provide insight into the link between phase coupling of RSA and SWS activity.

Another aspect of cardiorespiratory phase synchronization during sleep has been demonstrated by analysis using synchrograms (Bartsch et al., 2007, 2012). The cardiorespiratory synchronization index is quantified by the extent to which heartbeats are clustered at specific phases in the breathing cycle. Bartsch et al. (2012) observed a relatively low phase synchronization during REM sleep, higher synchronization during light sleep, and the highest synchronization during deep sleep. Again, considering the RSA as a major portion of heartbeat fluctuation, their observations of an increase in the degree of phase synchronization between respiration and heartbeat during NREM sleep is essentially equivalent to our observation that the phase coupling of RSA increased with an increase in δ-wave activity, as such a situation occurs during NREM.

We observed that the correlation between δ-wave activity and λ was comparable to the correlation between δ-wave activity and the HF_n_ index, but significantly higher than that the correlation between δ-wave activity and A_RSA_ in the Entire and F3_4 sleep periods. We cannot explain this observation physiologically but it may be related, at least in part, to the oscillatory behavior of A_RSA_. As shown in Figure 6A, the OS of the A_RSA_ was significantly lower than that for δ-wave, λ, and HF_n_. Because OS is indicative of the degree to which the ACF oscillating within a given frequency band corresponds to the sleep cycle, this indicates that the ACF oscillation of A_RSA_ is characterized by broad spectral distributions. In this study, A_RSA_ was calculated as the envelope of the RSA oscillation using Hilbert transform, where abrupt changes in RRI are captured in the envelope, which would lead to oscillatory behavior with a higher frequency than the sleep cycle in the ACF, and consequently result in a lower correlation between A_RSA_ and δ-wave activity.

Although the CCF_max_ between the δ-wave and HF_n_ was reduced in the L3_4 sleep period compared to the Entire and F3_4 sleep periods, CCF_max_ between the δ-wave and λ did not change considerably based on the sleep periods analyzed (Figure 6B). However, the relationship of the sleep cycles estimated from the ACFs of λ and the δ-wave diverged from the identity line in the L3_4 period. This might be due to the fact that SWS is shorter and sparser during later sleep cycles. We observed that δ-wave and λ periodicities were more prominent in the first half of the night, which is consistent with the widely-held view that most SWS occurs during the first 1-2 sleep cycles (Borbély and Achermann, 2005; Riedner et al., 2007). One proposed explanation for this trend is synaptic down-scaling (Tononi and Cirelli, 2006, 2014). During SWS, cortical networks oscillate between periods of high and low activity (corresponding to periods of depolarization and hyperpolarization, respectively). The repeated sequence of widespread membrane depolarization and hyperpolarization in a δ-frequency range favors processes of synaptic depression in the network. As a consequence of ongoing synaptic downscaling in a way that maintains synaptic homeostasis, SWS gradually decreases across the sleep period. Although this trend of SWS cycle could be captured by λ across nearly the entire sleep period, it should be noted that sensitivity of λ to predicting SWS with respect to the elapsed time of sleep was poor when prediction was performed at later stage of sleep period.

The mechanism responsible for alterations in the degree of phase coupling of RSA remains unclear. As is well known, RSA is predominantly mediated by respiratory gating of parasympathetic efferent activity to the heart (Eckberg, 2003). The transduction gain and delay of the parasympathetic nervous system have been shown to vary with change in f_R_; faster breathing cause an attenuation of A_RSA_ and an increasing delay (Hirsch and Bishop, 1981; Saul et al., 1989). We observed that the decrease in λ was accompanied by an increase in the variation of f_R_ (Table 1). Because λ represents the degree of phase synchronization between the oscillations, variations of f_R_ in a time window would lead to a decrease in λ. An alternative explanation for the observation of a lower λ during non-SWS period, when the δ-activity is reduced (sympathetic tone is dominant), is that the enhanced sympathetic nerve activity may modulate the transduction property of the vagal efferent nerve to the heart on a breath-to-breath basis. It has been suggested that neuromodulators released from sympathetic nerve terminals can exert an inhibitory action on the phasic vagal drive (Potter, 1985), which in turn may affect phase coupling of RSA in addition to causing a decrease in the amplitude of RSA. Given that sympathetic activation associated with sleep stage transitions leads to variability of the transduction property of the vagal nerve, an increase in the degree of phase coupling of RSA appears to be an indication of a predominance of vagal activity, and a well-adjusted cardiac and respiratory interaction that occurs during SWS period.

In consideration of our results, there are methodological concerns that warrant discussion. First, oronasal flow, which is commonly used for the sleep study, was not measured. We considered that wearing an oronasal mask may cause unnecessary stress on the subjects and RIP enables monitoring breathing activity in a more natural setting. Although there may be a phase delay between actual respiratory flow and RIP signal, this delay is not essential for the computation of λ, since λ measures a consistency of the relative phase difference between RSA and RIP signal in the given window. Second, subjects' age range was relatively large, which must be taken into consideration when interpreting the results. Age may be an important consideration because sleep architecture differs depending on age (Aström and Trojaborg, 1992; Landolt et al., 1996). It has been demonstrated that older adults experience more frequent awakening during sleep because SWS is thought to decline with age (Carrier et al., 2001). Because of small numbers of female and elderly subjects, we could not conduct a comparative analysis between younger and elderly subjects nor between genders. It should be noted that the present findings were from a hypothesis-driven study and a larger confirmatory study is needed to retain more statistical power. Third, an obvious limitation of the present study was the absence of polysomnographic recordings and therefore sleep staging. However, the aim of this study was to quantify the temporal relationship between phase coupling of RSA and δ-wave activity. A single Fp1 recording to derive brain activity was not a concern, because sleep scoring from frontopolar EEG recordings has shown good agreement with standard central EEG derivation (Lapinlampi and Himanen, 2004) and slow waves are most prevalent in frontal brain areas (Cajochen et al., 1999). However, it is uncertain how changes in λ might relate to sleep-stage transitions. We can only say that variation in λ is correlated with the preceding changes in δ-wave activity during sleep. Considering that sympathetic tone is activated during REM sleep and that the transition to NREM sleep induces a shift to a more predominate parasympathetic influence, as has been suggested previously (Berlad et al., 1993; Toscani et al., 1996; Monti et al., 2002), λ is expected to decrease during REM sleep, but increase depending on the degree of sleep depth. Changes in λ as they relate to sleep stages warrant further investigation.

In conclusion, we examined the dynamics of cardiorespiratory phase coupling during sleep in healthy humans, with a focus on the temporal association of brain slow wave activity. We have shown that the temporal dynamics of cardiorespiratory phase coupling, as assessed by phase coherence (λ) between RSA and respiration, is significantly associated with δ-wave activity, with λ preceding the change in δ-wave activity by ~3 min. The period of the cyclical change in λ was found to be close to that in the δ wave, by autocorrelation analysis, suggesting that λ may provide information on the SWS period, thereby complementing other noninvasive tools and diagnostic efforts. We believe that these findings will be useful in the evaluation of sleep cycles in terms of the period and rhythmicity of λ, as well as in the evaluation of the relationship between EEG activity and ANS function during sleep.

## Author contributions

KN and TS conceived of the research plan. KN assumed responsibility for the integrity of the data and the accuracy of the data analysis. He prepared the figures and drafted the manuscript. KN and TS reviewed the final manuscript.

### Conflict of interest statement

The authors declare that the research was conducted in the absence of any commercial or financial relationships that could be construed as a potential conflict of interest.

## Supplementary material

Figure S1 Identification of high δ-wave activity segments. The regions where the amplitude of δ-activity was above the mean amplitude of δ-activity in the entire night of sleep were identified as the high δ-wave activity segments. Red horizontal line shows the mean level of δ-wave amplitude. Note that the time courses for λ, HF_n_, and A_RSA_ were aligned with the respective T_d_ values observed in the CCFs with δ-wave, in order to compare the mean values of parameters between high and low δ-activity segments. Histograms in each variable are shown in right side.

## References

[B1] Aeschbach D. Borbély A. A. (1993). All night dynamics of human sleep EEG. J. Sleep Res. 2, 70–81. 10.1111/j.1365-2869.1993.tb00065.x 10607074

[B2] Aström C. Trojaborg W. (1992). Relationship of age to power spectrum analysis of EEG during sleep. J. Clin. Neurophysiol. 9, 424–430. 10.1097/00004691-199207010-00010 1517410

[B3] Bartsch R. P. Kantelhardt J. W. Penzel T. Havlin S. (2007). Experimental evidence for phase synchronization transitions in human cardio-respiratory system. Phys. Rev. Lett. 98:054102 10.1103/PhysRevLett.98.054102 17358862

[B4] Bartsch R. P. Schumann A. Y. Kantelhardt J. W. Penzel T. Ivanov P. Ch. (2012). Phase transitions in physiologic coupling. Proc. Natl. Acad. Sci. U.S.A. 109, 10181–10186. 10.1073/pnas.1204568109 22691492 PMC3387128

[B5] Berlad I. Shlitner A. Ben-Haim S. Lavie P. (1993). Power spectrum analysis and heart rate variability in stage 4 and REM sleep: evidence for state-specific changes in autonomic dominance. J. Sleep Res. 2, 88–90. 10.1111/j.1365-2869.1993.tb00067.x 10607076

[B6] Berntson G. G. Bigger J. T. Eckberg D. L. Grossman P. Kaufmann P. G. Malik M.. (1997). Heart rate variability: origins, methods, and interpretive caveats. Psychophysiology, 34, 623–648. 10.1111/j.1469-8986.1997.tb02140.x 9401419

[B7] Billman G. E. (2013). The LF/HF ratio does not accurately measure cardiac sympatho-vagal balance. Front. Physiol. 4:26 10.3389/fphys.2013.00026 23431279 PMC3576706

[B8] Borbély A. A. Achermann P. (2005). Sleep homeostasis and models of sleep regulation, in Principles and Practice of Sleep Medicine, eds Kryger M. H. Roth T. Dement W. C. (Philadelphia, PA: Elsevier Inc.), 405–417.

[B9] Born J. Muth S. Fehm H. L. (1988). The significance of sleep onset and slow wave sleep for nocturnal release of growth hormone and cortisol. Psychoneuroendocrinology 13, 233–243. 10.1016/0306-4530(88)90021-2 3406323

[B10] Brandenberger G. Ehrhart J. Buchheit M. (2005). Sleep stage 2: an electroencephalographic, autonomic, and hormonal duality. Sleep 28, 1535–1540. 10.1093/sleep/28.12.1535 16408412

[B11] Brandenberger G. Ehrhart J. Piquard F. Simon C. (2001). Inverse coupling between ultradian oscillations in delta wave activity and heart rate variability during sleep. Clin. Neurophysiol. 112, 992–996. 10.1016/S1388-2457(01)00507-7 11377256

[B12] Burgess H. J. Holmes A. L. Dawson D. (2001). The relationship between slow-wave activity, body temperature, and cardiac activity during nighttime sleep. Sleep 24, 343–349. 10.1093/sleep/24.3.343 11322718

[B13] Cabiddu R. Cerutti S. Viardot G. Werner S. Bianchi A. M. (2012). Modulation of the sympatho-vagal balance during sleep: frequency domain study of heart rate variability and respiration. Front. Physiol. 3:45. 10.3389/fphys.2012.00045. 22416233 PMC3299415

[B14] Cajochen C. Foy R. Dijk D. J. (1999). Frontal predominance of a relative increase in sleep delta and theta EEG activity after sleep loss in humans. Sleep Res. Online 2, 65–69. 11382884

[B15] Carrier J. Land S. Buysse D. J. Kupfer D. J. Monk T. H. (2001). The effects of age and gender on sleep EEG power spectral density in the middle years of life (ages 20–60 years old). Psychophysiology 38, 232–242. 10.1111/1469-8986.3820232 11347869

[B16] Daubechies I. (1990). The wavelet transform, time-frequency localization and signal analysis. IEEE Trans. Info. Theory 36, 961–2005.

[B17] Eckberg D. L. (2003). The human respiratory gate. J. Physiol. 548, 339–352. 10.1113/jphysiol.2002.037192 12626671 PMC2342859

[B18] Fung M. Peters K. Redline S. Ziegler M. G. Ancoli-Israel S. Barrett-Connor E.. (2011). Decreased slow wave sleep increases risk of developing hypertension in elderly men. Hypertension 58, 596–603. 10.1161/HYPERTENSIONAHA.111.174409 21876072 PMC3176739

[B19] Gais S. Born J. (2004). Low acetylcholine during slow-wave sleep is critical for declarative memory consolidation. Proc. Natl. Acad. Sci. U.S.A. 101, 2140–2144. 10.1073/pnas.0305404101 14766981 PMC357065

[B20] Goldstein D. S. Bentho O. Park M. Y. Sgarabi Y. (2011). Low-frequency power of heart rate variability is not a measure of cardiac sympathetic tone but may be a measure of modulation of cardiac autonomic outflows by baroreflexes. Exp. Physiol. 96, 1255–1261. 10.1113/expphysiol.2010.056259 21890520 PMC3224799

[B21] Hirsch J. A. Bishop B. (1981). Respiratory sinus arrhythmia in humans: how breathing patterns modulates heart rate. Am. J. Physiol. Heart Circ. Physiol. 241, H620–H629. 10.1152/ajpheart.1981.241.4.H620 7315987

[B22] Iber C. Ancoli-Israel S. Chesson A. Quan S. F. (2007). The AASM Manual for the Scoring of Sleep and Associated Events: Rules, Terminology and Technical Specifications. 1st Edn Westchester, IL: American Academy of Sleep Medicine.

[B23] Jurysta F. van de Borne P. Migeotte P.-F. Dumont M. Lanquart J.-P. Degaute J.-P.. (2003). A study of the dynamic interactions between sleep EEG and heart rate variability in healthy young men. Clin. Neurophysiol. 114, 2146–2155. 10.1016/S1388-2457(03)00215-3 14580613

[B24] Jurysta F. van de Borne P. Migeotte P.-F. Dumont M. J.-P. Degaute J.-P. Linkowski P. (2006). The link between cardiac autonomic activity and sleep delta power is altered in men with sleep apnea hypopnea syndrome. Am. J. Physiol. Regul. Integr. Comp. Physiol. 291, R1165–R1171. 10.1152/ajpregu.00787.2005 16675631

[B25] Landolt H. P. Dijk D. J. Achermann P. Borbely A. A. (1996). Effects of age on the sleep EEG: slow wave activity and spindle frequency activity in young and middle aged men. Brain Res. 738, 205–212. 10.1016/S0006-8993(96)00770-6 8955514

[B26] Lapinlampi A. M. Himanen S. L. (2004). Sleep staging with frontopolar EEG derivation. Sleep Hypn. 6, 48–54.

[B27] Long X. Arends B. J. Aarts R. M. Haakma R. Fonseca P. Rolink J. (2015).Time delay between cardiac and brain activity during sleep transitions. Appl. Phys. Lett. 106, 143702-1-4. 10.1063/1.4917221

[B28] Magnin M. Rey M. Bastuji H. Guillemant P. Mauguiere F. Garcia-Larrea L. (2010). Thalamic deactivation at sleep onset precedes that of the cerebral cortex in humans. Proc. Natl. Acad. Sci. U.S.A. 107, 3829–3833. 10.1073/pnas.0909710107 20142493 PMC2840430

[B29] Merica H. Gaillard J. M. (1985). Statistical description and evaluation of the interrelationships of standard sleep variables for normal subjects. Sleep 8, 261–273. 10.1093/sleep/8.3.261 4048742

[B30] Miyashita T. Ogawa K. Itoh H. Arai Y. Ashidagawa M. Uchiyama M.. (2003). Spectral analyses of electroencephalography and heart rate variability during sleep in normal subjects. Auton. Neurosci. 103, 114–120. 10.1016/S1566-0702(02)00259-X 12531405

[B31] Monti A. Medigue C. Nedelcoux H. Escourrou P. (2002). Autonomic control of the cardiovascular system during sleep in normal subjects. Eur. J. Appl. Physiol. 87, 174–181. 10.1007/s00421-002-0597-1 12070629

[B32] National Sleep Foundation (2006). The Sleep-Wake Cycle: Its Physiology and Impact on Health, Arlington, TX: NSF.

[B33] Niizeki K. Saitoh T. (2012). Incoherent oscillations of respiratory sinus arrhythmia during acute mental stress in humans. Am. J. Physiol. Heart Circ. Physiol. 302, H359–H367. 10.1152/ajpheart.00746.2011 22037190

[B34] Niizeki K. Saitoh T. (2016). Analysis of cardiorespiratory phase coupling and cardiovascular autonomic responses during food ingestion. Physiol. Behav. 159, 1–13. 10.1016/j.physbeh.2016.03.004 26969519

[B35] Otzenberger H. Gronfier C. Simon C. Charloux A. Ehrhart J. Piquard F.. (1998). Dynamic heart rate variability: a tool for exploring sympathovagal balance continuously during sleep in men. Am. J. Physiol. Heart Circ. Physiol. 275, H946–H950. 10.1152/ajpheart.1998.275.3.H946 9724299

[B36] Otzenberger H. Simon C. Gronfier C. Brandenberger G. (1997). Temporal relationship between dynamic heart rate variability and electroencephalographic activity during sleep in man. Neurosci. Lett. 229, 173–176. 10.1016/S0304-3940(97)00448-5 9237486

[B37] Penzel T. Kantelhardt J. W. Bartsch R. P. Riedl M. Kraemer J. F. Wessel N. (2016) Modulations of heart rate, ECG, cardio-respiratory coupling observed in polysomnography. Front. Physiol. 7:460 10.3389/fphys.2016.00460. 27826247 PMC5078504

[B38] Potter E. K. (1985). Prolonged non-adrenergic inhibition of cardiac vagal action following sympathetic stimulation: neuromodulation by neuropeptide Y? Neurosci. Lett. 54, 117–121. 10.1016/S0304-3940(85)80065-3 2859560

[B39] Randall D. C. Brown D. R. Raisch R. M. Yingling J. D. Randall W. C. (1991) SA nodal parasympathectomy delineates autonomic control of heart rate power spectrum. Am. J. Physiol. Heart Circ. Physiol. 260, H985–H988. 10.1152/ajpheart.1991.260.3.H985 1672056

[B40] Riedner B. A. Vyazovskiy V. V. Huber R. Massimini M. Esser S. Murphy M. (2007). Sleep homeostasis and cortical synchronization: III. A high-density EEG study of sleep slow waves in humans. Sleep. 30, 1643–1657. 10.1093/sleep/30.12.1643 18246974 PMC2276133

[B41] Saul J. P. Berger R. D. Chen M. H. Cohen R. J. (1989). Transfer function analysis of autonomic regulation 2. Respiratory sinus arrhythmia. Am. J. Physiol. Heart Circ. Physiol. 256, H153–H161. 10.1152/ajpheart.1989.256.1.H153 2912177

[B42] Shinar Z. Akselrod S. Dagan Y. Baharav A. (2006). Autonomic changes during wake-sleep transition: a heart rate variability based approach. Auton. Neurosci. 130, 17–27. 10.1016/j.autneu.2006.04.006 16759916

[B43] Somers V. K. Dyken M. E. Clary M. P. Abboud F. M. (1995). Sympathetic neural mechanism in obstractive sleep apnea. J. Clin. Invest. 96, 1897–1904. 10.1172/JCI118235 7560081 PMC185826

[B44] Taheri S. Lin L. Austin D. Young T. Mignot E. (2004). Short sleep duration is associated with reduced leptin, elevated ghrelin, and increased body mass index. PLoS Med. 1:e62 10.1371/journal.pmed.0010062 15602591 PMC535701

[B45] Tasali E. Leproult R. Ehrmann D. A. Van Cauter E. (2008). Slow-wave sleep and the risk of type 2 diabetes in humans. Proc. Natl. Acad. Sci. U.S.A. 105, 1044–1049. 10.1073/pnas.0706446105 18172212 PMC2242689

[B46] Thomas R. J. Mietus J. E. Peng C.-K. Guo D. Gozal D. Montgomery-Down H.. (2014). Relationship between delta power and the electrocardiogram-derived cardiopulmonary spectrogram: possible implications for assessing the effectiveness of sleep. Sleep Med. 15, 125–131. 10.1016/j.sleep.2013.10.002 24269134 PMC4114218

[B47] Tobaldini E. Nobili L. Strada S. Casali K. R. Braghiroli A. Montano N. (2013). Heart rate variability in normal and pathological sleep. Front. Physiol. 4:294. 10.3389/fphys.2013.00294 24137133 PMC3797399

[B48] Tononi G. Cirelli C. (2006). Sleep function and synaptic homeostasis. Sleep Med. Rev. 10, 49–62. 10.1016/j.smrv.2005.05.002 16376591

[B49] Tononi G. Cirelli C. (2014). Sleep and the price of plasticity: from synaptic and cellular homeostasis to memory consolidation and integration. Neuron 81, 12–34. 10.1016/j.neuron.2013.12.025 24411729 PMC3921176

[B50] Toscani L. Gangemi P. F. Parigi A. Silipo R. Ragghianti P. Sirabella E.. (1996). Human heart rate variability and sleep stages. Ital. J. Neurol. Sci. 17, 437–439. 10.1007/BF01997720 8978452

[B51] Vaughn B. V. Quint S. R. Messenheimer J. A. Robertson K. R. (1995). Heart rate variability in sleep. Electroencep. Cli. Neurophysiol. 94, 155–162. 10.1016/0013-4694(94)00270-U 7536150

