# Supplementary figures and images for: Association Between Phase Coupling of Respiratory Sinus Arrhythmia and Slow Wave Brain Activity During Sleep
Source: Front Physiol. 2018 Sep 25;9:1338. doi: 10.3389/fphys.2018.01338 (PMC6167474; doi:10.3389/fphys.2018.01338)

## Slide 1
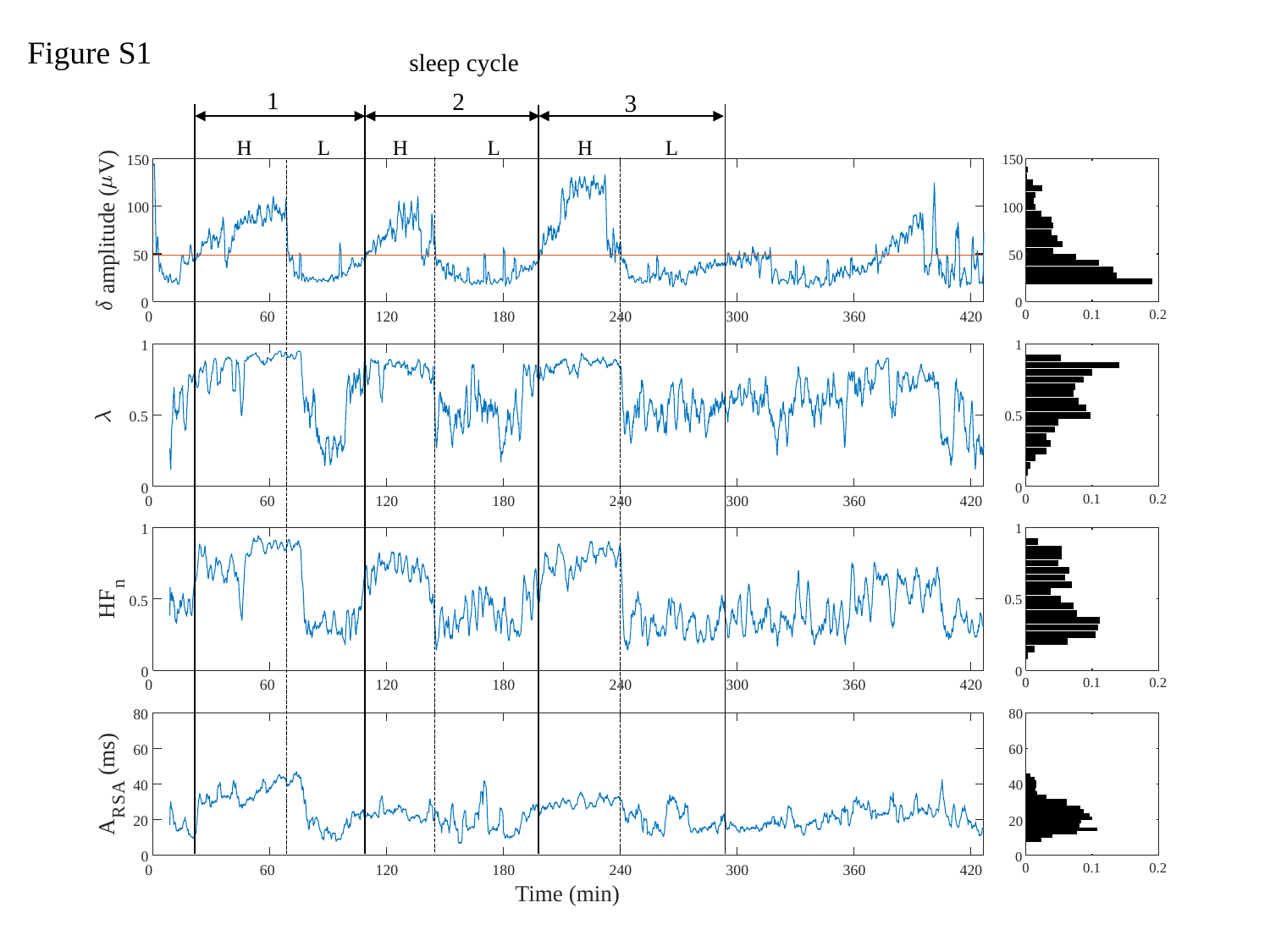

Figure S1
sleep cycle
1
2
3
H
H
L
H
L
L

Supplement: Figure S1 — Identification of high δ-wave activity segments. The regions where the amplitude of δ-activity was above the mean amplitude of δ-activity in the entire night of sleep were identified as the high δ-wave activity segments. Red horizontal line shows the mean level of δ-wave amplitude. Note that the time courses for λ, HFn, and ARSA were aligned with the respective Td values observed in the CCFs with δ-wave, in order to compare the mean values of parameters between high and low δ-activity segments. Histograms in each variable are shown in right side. [file Presentation_1.PPTX]
